# Male Breast Cancer: Results of the Application of Multigene Panel Testing to an Italian Cohort of Patients

**DOI:** 10.3390/diagnostics10050269

**Published:** 2020-04-30

**Authors:** Gianluca Tedaldi, Michela Tebaldi, Valentina Zampiga, Ilaria Cangini, Francesca Pirini, Elisa Ferracci, Rita Danesi, Valentina Arcangeli, Mila Ravegnani, Giovanni Martinelli, Fabio Falcini, Paola Ulivi, Daniele Calistri

**Affiliations:** 1 Biosciences Laboratory, Istituto Scientifico Romagnolo per lo Studio e la Cura dei Tumori (IRST) IRCCS, 47014 Meldola, Italy; valentina.zampiga@irst.emr.it (V.Z.); ilaria.cangini@irst.emr.it (I.C.); francesca.pirini@irst.emr.it (F.P.); elisa.ferracci@irst.emr.it (E.F.); daniele.calistri@irst.emr.it (D.C.); 2 Biostatistics and Clinical Trials Unit, Istituto Scientifico Romagnolo per lo Studio e la Cura dei Tumori (IRST) IRCCS, 47014 Meldola, Italy; michela.tebaldi@irst.emr.it; 3 Romagna Cancer Registry, Istituto Scientifico Romagnolo per lo Studio e la Cura dei Tumori (IRST) IRCCS, 47014 Meldola, Italy; rita.danesi@irst.emr.it (R.D.); mila.ravegnani@irst.emr.it (M.R.); fabio.falcini@irst.emr.it (F.F.); 4 Department of Medical Oncology, Ospedale Infermi, 47923 Rimini, Italy; valentina.arcangeli@auslromagna.it; 5 Department of Medical Oncology, Istituto Scientifico Romagnolo per lo Studio e la Cura dei Tumori (IRST) IRCCS, 47014 Meldola, Italy; giovanni.martinelli@irst.emr.it

**Keywords:** male breast cancer, next-generation sequencing, cancer predisposition, *BRCA1/2* genes, hereditary cancer, multigene panel testing, multiplex ligation-dependent probe amplification

## Abstract

Male breast cancer (MBC) is a rare tumor, accounting for less than 1% of all breast cancers. In MBC, genetic predisposition plays an important role; however, only a few studies have investigated in depth the role of genes other than *BRCA1* and *BRCA2*. We performed a Next-Generation Sequencing (NGS) analysis with a panel of 94 cancer predisposition genes on germline DNA from an Italian case series of 70 patients with MBC. Moreover, we searched for large deletions/duplications of *BRCA1/2* genes through the Multiplex Ligation-dependent Probe Amplification (MLPA) technique. Through the combination of NGS and MLPA, we identified three pathogenic variants in the *BRCA1* gene and six in the *BRCA2* gene. Besides these alterations, we found six additional pathogenic/likely-pathogenic variants in *PALB2*, *CHEK2*, *ATM*, *RAD51C*, *BAP1* and *EGFR* genes. From our study, *BRCA1* and *BRCA2* emerge as the main genes associated with MBC risk, but also other genes seem to be associated with the disease. Indeed, some of these genes have already been implicated in female breast cancer predisposition, but others are known to be involved in other types of cancer. Consequently, our results suggest that novel genes could be involved in MBC susceptibility, shedding new light on their role in cancer development.

## 1. Introduction

Breast cancer (BC) is the first tumor for incidence and mortality in women [1], but it can also affect men. Indeed, male breast cancer (MBC) represents less than 1% of all BCs [2]. However, being a rare and poorly known disease, it is often diagnosed at later stages with the consequence of a poor prognosis [3].

Similarly to female breast cancer (FBC), genetic predisposition is an important risk factor in MBC [4,5]. *BRCA1* [6] and *BRCA2* [7] are the genes best known for their involvement in BC predisposition in both females and males [8], with an MBC risk that has been estimated of 1.2% and 6.8% by the age of 70 for carriers of variants in *BRCA1* and *BRCA2*, respectively [9].

In recent years, thanks to the wide use of Next-Generation Sequencing (NGS), the number of genes suspected to be involved in cancer predisposition has dramatically increased [10]. This is true especially for cancers with a strong hereditary component such as FBC, in which several studies have investigated the role of genes other than *BRCA1/2* [11,12,13,14,15,16,17,18,19,20]. Some studies have highlighted the role of genes such as *PALB2*, *ATM*, *CHEK2*, *FANCM*, *PTEN*, *APC* and *MUTYH* in MBC [21,22,23,24,25,26,27,28], thus excluding others, such as *BRIP1* and *RAD51C* [29,30,31].

Within this complex scenario, the aim of our study was the application of multigene panel testing (MGPT) in a series of patients with MBC, in order to assess the presence of *BRCA1/2* variants and identify new genes involved in the predisposition to MBC.

## 2. Materials and Methods

### 2.1. Ethics Statement

The study was conducted in accordance with ethical standards, the Declaration of Helsinki and national and international guidelines, and was approved by our local ethics committee (CE IRST IRCCS-AVR, protocol 2207/2012). All the patients enrolled in the study have signed an informed consent for the genetic analyses and for the use of the results for research purposes. 

### 2.2. Patients’ Selection

The patients have been selected through an appropriate oncologic genetic counseling according to the Hereditary Breast Cancer Protocol of the Emilia-Romagna region [32]. During the genetic counseling, the personal and family history of cancer was collected and verified by our medical geneticists. According to the FONCaM guidelines used in the protocol [33], a diagnosis of MBC at any age is sufficient to proceed with the *BRCA1/2* genetic test, regardless of family history of breast and ovarian cancers (BC/OC). Through this procedure, we selected 70 Italian patients with a diagnosis of MBC.

### 2.3. Sample Collection and DNA Extraction

Peripheral blood samples were collected from the 70 MBC patients and stored at −80 °C until the genetic analyses. Genomic DNA was extracted from blood using QIAamp DNA Mini Kit (Qiagen, Hilden, Germany) and quantified using Qubit fluorometer and Qubit dsDNA BR Assay Kit (Thermo Fisher Scientific, Waltham, MA, USA).

### 2.4. Next-Generation Sequencing (NGS)

We used 50 ng of DNA from each sample to create libraries following the Trusight Rapid Capture protocol (Illumina, San Diego, CA, USA). Libraries were enriched for the regions of interest with the Trusight Cancer panel (Illumina), which contains probes targeting the coding regions of 94 genes involved in hereditary cancer (Table 1).

Libraries were sequenced on the MiSeq platform (Illumina) with MiSeq Reagent Kit v2 configured 2 × 150 cycles, according to the manufacturer’s instructions.

All deleterious variants (classes 4–5) identified in *BRCA1/2* genes were confirmed through Sanger sequencing following a custom protocol previously described [15]. The same procedure was performed for the *BRCA1/2* coding regions with a coverage < 50×. The deleterious variants identified in other genes were confirmed through a second NGS analysis with the same kit and protocol.

### 2.5. Bioinformatics Analysis

The bioinformatics analysis of NGS results was performed with a customized pipeline described in our previous studies [15,34]. The fastq files have been aligned with BWA software [35] to the reference genome, version UCSC-Build37/hg19. Reads were remapped against the Trusight Cancer Panel reference, removed from duplicates and then realigned for germline indels using GATK indelRealigner. Finally, variants were called using Unified Genotyper of GATK software, version 3.2.2 [36]. Genomic and functional annotations of detected variants were made by Annovar [37].

### 2.6. Multiplex Ligation-Dependent Probe Amplification (MLPA)

The presence of large deletions/duplications of *BRCA1/2* genes, not detectable with the Trusight protocol, was assessed by Multiplex Ligation-dependent Probe Amplification (MLPA) technique with the P002-BRCA1 and P045-BRCA2/CHEK2 kits (MRC Holland, Amsterdam, The Netherlands). The large *BRCA1* deletion identified by MLPA was confirmed through the P087-BRCA1 confirmation kit (MRC Holland). Coffalyser software (MRC Holland) was used for the quantitative analysis of the electropherograms.

### 2.7. Variant Classification

All the genetic variants identified were divided into five classes according to the International Agency for Research on Cancer (IARC) [38]: Benign Variant (BV-class 1), Likely-Benign Variant (LBV-class 2), Variant of Uncertain Significance (VUS-class 3), Likely-Pathogenic Variant (LPV-class 4) and Pathogenic Variant (PV-class 5). The process of variant classification was performed in accordance with the guidelines of the American College of Medical Genetics (ACMG) [39].

In particular, for *BRCA1/2* variants, we used different BRCA-specific databases [40,41,42,43,44,45]. The variants of other genes were classified using comprehensive variant databases [46,47,48].

## 3. Results

### 3.1. Patient Characteristics

All 70 patients selected for the study had MBC between 36 and 87 years of age, with an average age at cancer onset of 63.8 years. Among the patients, 2/70 (2.9%) had a second contralateral MBC and 16/70 (22.9%) had a second non-BC malignancy.

Regarding the histopathological classification [49], 56/70 MBCs (80.0%) were infiltrating ductal carcinomas (IDC-8500/3), 9/70 (12.9%) were ductal carcinomas in situ (DCIS-8500/2), 3/70 (4.3%) were infiltrating papillary carcinomas (IPC-8503/3) and 2/70 (2.9%) were papillary carcinomas in situ (PCIS-8503/2).

As far as it concerns the family history of cancer, 24/70 patients (34.3%) had I- and/or II-degree relatives with BC/OC and 17/70 patients (24.3%) had I- and/or II-degree relatives with other cancers. In particular, 3/70 patients (4.3%) had a family history of MBC among I-degree relatives.

### 3.2. Pathogenic and Likely-Pathogenic Variants in BRCA1/2 Genes

Since *BRCA1* and *BRCA2* variants are the most common alterations in BC patients, we initially focused on these genes.

The NGS analysis allowed us to detect eight pathogenic variants (PVs) in *BRCA1/2* genes. In particular, we identified the c.4964_4982del and the c.5266dupC variants in *BRCA1* gene, both already reported [46] and classified as pathogenic [47]. In *BRCA2* gene, we identified five PVs in six patients, c.1238delT, c.1813delA, c.3195_3198delTAAT, c.5073dupA in single patients and c.6039delA in two unrelated patients. In addition, these variants were already reported [46] and classified as pathogenic [47].

The MLPA analysis revealed an additional PV in *BRCA1* gene, a large deletion covering exons 1 and 2. However, the MLPA technique does not give information about the exact location of the breakpoints and, consequently, the variant has been classified as c.-113-?_80+?del. This variant is not reported in the databases, but the deletion of the transcription start site and the first two exons certainly has a deleterious effect on the gene transcription. Moreover, large deletions involving these two exons have been previously described as pathogenic in BC/OC patients [50,51,52,53,54,55,56,57]. 

**Figure 1:**
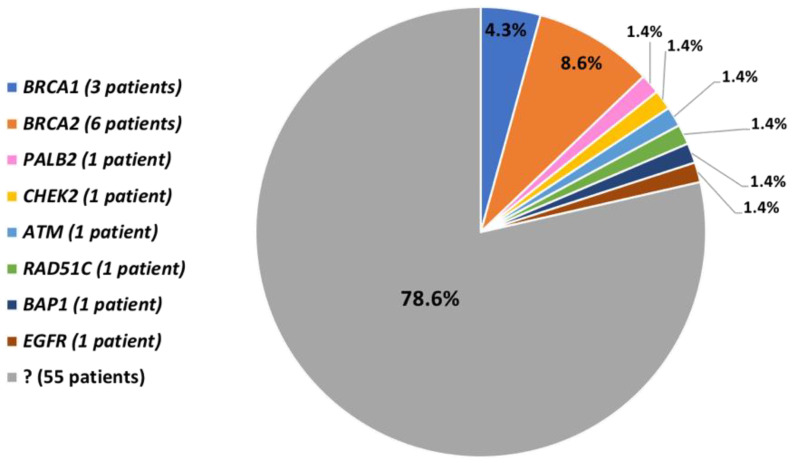
Pie chart showing the fraction of cases with/without PVs/LPVs; the number of variant carriers is reported between brackets.

**Figure 2:**
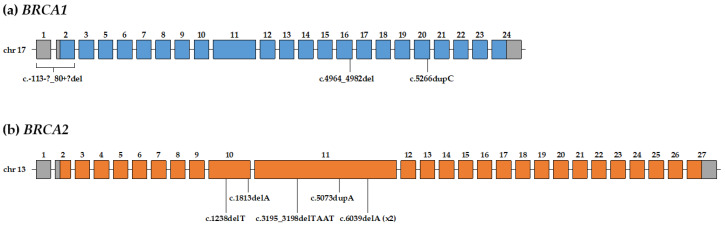
(**a**) Schematic representation of the *BRCA1* gene (transcript NM_007294) and localization of the three PVs identified in our cohort; (**b**) schematic representation of the *BRCA2* gene (transcript NM_000059) and localization of the six PVs identified in our cohort. Exons are represented with grey boxes for non-coding exons and with blue and orange boxes for coding exons of *BRCA1* and *BRCA2*, respectively; the exon numbering has been reported above the exons (the traditional exon numbering of *BRCA1* gene lacks exon four that has not been represented).

Overall, we identified 9/70 patients (12.9%) with *BRCA1/2* PVs (Figure 1). All the *BRCA1/2* PVs identified in our cohort are reported in Figure 2 and Table 2 with the corresponding patient characteristics. In particular, 8/9 patients had an IDC and 1/9 had a DCIS. The average age at MBC onset was 62.5 years. Regarding the family history of cancer, 5/9 patients (55.6%) had I- and/or II-degree relatives with BC/OC and 3/9 patients (33.3%) had I- and/or II-degree relatives with other cancers.

### 3.3. Pathogenic and Likely-Pathogenic Variants in other Genes

After the assessment of the *BRCA1/2* mutation status of our patients, we searched for PVs and likely-pathogenic variants (LPVs) in the other 92 genes of the panel. We detected six additional PVs/LPVs of six different genes in 6/70 patients (8.6%) (Figure 1). In particular, we identified the c.8319_8323dupTGTCC variant in *ATM* gene, the c.1110_1116delCATGCAG variant in *BAP1* gene, the c.1100delC variant in *CHEK2* gene, the c.3538_3541delGAAG in the *EGFR* gene, the c.73A>T variant in *PALB2* gene and the c.181_182delCT in *RAD51C* gene. The variants in *ATM*, *CHEK2*, *PALB2* and *RAD51C* genes were already reported [46] and classified as pathogenic [47]. On the opposite, the variant in *EGFR* gene was reported only in dbSNP without classification [46] and the variant in *BAP1* gene was novel. According to the guidelines [39], we classified these two alterations as LPVs.

All the PVs/LPVs identified in genes other than *BRCA1/2* are reported in Table 3 with the corresponding patient characteristics. In particular, 5/6 patients had an IDC and 1/6 had a DCIS. The average age at MBC onset was 55.8 years. Regarding the family history of cancer, 2/6 patients (33.3%) had I- and/or II-degree relatives with BC/OC and 2/6 patients (33.3%) had I- and/or II-degree relatives with other cancers.

### 3.4. Patients without Pathogenic and Likely-Pathogenic Variants

In the remainder of our cohort, i.e., 55 patients (78.6%), we did not find any PV/LPV neither in *BRCA1/2* genes nor in the other 92 genes of the panel (Figure 1).

All these patients had MBC with an average age at cancer onset of 64.9 years. In particular, 43/55 patients had an IDC, 7/55 had a DCIS, 3/55 had an IPC and 2/55 had a PCIS. Regarding the family history of cancer, 17/55 patients (30.9%) had I- and/or II-degree relatives with BC/OC and 12/55 patients (21.8%) had I- and/or II-degree relatives with other cancers.

## 4. Discussion

MBC is a rare disease but deserves attention in terms of diagnosis and treatment as clinicians tend to follow the guidelines that have been developed for FBC management [58]. In this scenario, the research on the risk factors associated with MBC, in particular the genetic predisposition, is a key element in the prevention and early diagnosis of the disease.

In the present study, we performed an NGS analysis on an Italian cohort of 70 patients with MBC with a panel of 94 genes. At the same time, we conducted an MLPA analysis on *BRCA1/2* genes in order to identify also variants that are not easily detectable by NGS.

Among 70 cases of MBC, we detected *BRCA1/2* PVs in nine patients. *BRCA1* and *BRCA2* genes are known since a long time for their involvement in high risk of BC, also in male variant carriers. In particular, germline PVs/LPVs in the *BRCA1* gene are associated with a 57%–65% and 1.2% risk of developing BC in females and males, respectively, by the age of 70 [9,59,60,61]. In addition, *BRCA1* alterations have been associated with an increased risk of colon cancer [62], prostate cancer [63] and pancreatic cancer [64,65]. On the contrary, germline PVs/LPVs in the *BRCA2* gene are associated with a 45%–55% and 6.8% risk of developing BC for females and males, respectively, by the age of 70 [9,59,60,61]. In addition, *BRCA2* alterations have been associated with an increased risk of prostate cancer [66], pancreatic cancer [65,67], and uveal melanoma [68,69]. For these reasons, men with *BRCA1/2* PVs/LPVs should undergo clinical breast examination every 6–12 months, starting at the age of 35 [70], and annual prostate cancer screening, starting at the age of 40 (in particular in *BRCA2* variant carriers) [71], whereas screening for melanoma and pancreatic cancer should be evaluated on the basis of family history [70]. Overall, we detected a 12.9% of MBC patients with *BRCA1/2* alterations with an average age at onset of 62.5 years. This result is in accordance with the findings of other authors [24,72,73,74] and confirms further the high risk of BC also for male carriers, in particular *BRCA2* variant carriers. As expected, the majority of these cases have a positive family history for BC/OC. Indeed, the genetic test on the consenting relatives allowed us to detect several carriers of *BRCA1/2* alterations, that have been addressed to a surveillance protocol according to the guidelines [32,33].

In addition to *BRCA1/2* variants, we identified four PVs in *PALB2*, *ATM*, *CHEK2* and *RAD51C*, classified as moderate penetrance genes for the risk of BC and/or OC [5] and two LPVs in unexpected genes, such as *BAP1* and *EGFR*. The patients in which we detected PVs/LPVs in genes other than *BRCA1/2* have an average age at MBC onset (55.8 years), that is lower than the age of MBC patients without PVs/LPVs (64.9 years) and also than the age of MBC patients with *BRCA1/2* PVs (62.5 years). This result suggests that, besides *BRCA1* and *BRCA2*, the genetic predisposition to MBC is associated with a plethora of genes, some of which are known to be linked to BC risk and others are novel in the field of hereditary BC.

In particular, the *PALB2* gene, encoding a protein that interacts with BRCA2 during the homologous recombination, is the most promising gene that emerges from NGS studies on BC/OC predisposition [15,75,76,77]. *PALB2* alterations have also been reported recurrently in MBC patients [23,24,27]. Regarding the penetrance, a recent study, conducted on 524 families with *PALB2* variants, reported a BC risk of 53% for females and 1% for males by the age of 80 [21]. The same study also reported an increased risk for OC and pancreatic cancer, which has been estimated at 5% and 2%–3%, respectively, by the age of 80. Our patient with the *PALB2* alteration had an IDC at 75 years old and reported a family history of cancer, with the mother deceased for BC at 60 years old. The genetic analysis on the relatives showed that the variant is carried also by his sister, who is healthy and now under a surveillance program for BC risk. Our result confirms further the important role of *PALB2* gene in MBC predisposition and highlights the importance of developing a surveillance protocol for male carriers, since the current guidelines give suggestions only for the management of female carriers [70].

The *CHEK2* gene encodes a tumor suppressor protein involved in DNA damage repair and its germline alterations are associated with an increased BC risk for female carriers [78,79,80,81], which is estimated to be 20%–44% during lifetime [82,83,84]. In addition to BC, *CHEK2* PVs/LPVs have also been associated with other cancers [85], including prostate [86,87,88], colorectal [89], and gastric cancers [90]. In particular, the *CHEK2* variant c.1100delC, detected in one of our MBC patients, is associated with a two- to three-fold increase in BC risk in women and a ten-fold increase of risk in men [91,92,93]. The patient had an IDC at 36 years old and reported a family history of cancer, in particular, the father with prostate cancer at 70 years and the paternal grandfather with non-Hodgkin lymphoma at 85 years. Unexpectedly, the genetic test on the relatives showed that the variant was harbored by his mother and also by his brother, who are both healthy. This result confirms the moderate penetrance of this *CHEK2* variant but, at the same time, suggests further that *CHEK2* alterations are associated with BC risk also in men.

We identified also a PV in the *ATM* gene, encoding a protein involved in DNA repair and cell cycle control, whose germline alterations are associated with an increased BC risk for women, which is estimated to be 15%–60% during lifetime [94,95,96,97,98,99]. *ATM* alterations have been previously reported in MBC [100,101]; however, some studies detected no increased risk of MBC for *ATM* variant carriers [24,72]. Our patient who carried the *ATM* variant had an IDC at 38 years and reported a family history of cancer, in particular, the father deceased for melanoma at 65 years. The very young age at cancer onset of our case and the recurrent detection of *ATM* deleterious alterations in MBC patients suggest that the involvement of this gene in the predisposition to the disease should be further investigated in larger case series.

We also found a PV in *RAD51C* gene, encoding a protein involved in homologous recombination. Germline alterations of the *RAD51C* gene have been associated with an increased OC risk, whereas the BC risk for variant carriers is controversial [102,103,104,105]. Regarding MBC, *RAD51C* variants were initially excluded for the BC risk for males [31] but, more recently, a study on a large cohort of MBC patients detected PVs in *RAD51C* and *RAD51D* genes, encoding proteins of the same complex [24]. Our patient carrying the *RAD51C* alteration had an IDC at 59 years old and had a sister deceased for BC at 55 years. Our finding reinforces a possible role of *RAD51C* gene alterations in BC predisposition, even in male carriers, and confirms the rationale of including this gene in a panel for the assessment of BC risk.

Moreover, we identified the c.1110_1116delCATGCAG in the *BAP1* gene, encoding a nuclear ubiquitin carboxy-terminal hydrolase that contains binding domains for BRCA1, BARD1 and HCFC1 proteins [106]. Germline deleterious variants in the *BAP1* gene are associated with the BAP1 tumor predisposition syndrome, an autosomal dominant condition characterized by an increased risk for atypical Spitz tumors and other types of cancer, such as uveal melanoma, malignant mesothelioma, cutaneous melanoma, clear cell renal cell carcinoma, and basal cell carcinoma [107]. However, *BAP1* germline alterations have also been reported in patients with BC and seem to be associated with an increased risk of BC [108,109,110,111,112], even if further studies are needed to confirm this link. Besides this, *BAP1* somatic mutations have been identified in sporadic BCs [106,113] and the involvement of BAP1 protein in the BRCA pathway supports the role of this protein in BC development. Indeed, the variant identified in our MBC patient is a frameshift deletion that generates a premature stop codon in the middle of the transcript, destroying completely the BRCA1 binding domain. The patient had an IDC at 65 years, he had no family history of cancer in I- and II-degree relatives but reported a female cousin deceased for BC before the age of 40. Although we were not able to verify the segregation of the disease with the genetic alteration, taking into account all the above considerations, we can conclude that *BAP1* is an emerging gene in the predisposition to BC and that also male carriers seem to have an increased BC risk.

The most unexpected result we obtained was the identification of a germline frameshift deletion in *EGFR* gene, encoding the Epidermal Growth Factor Receptor. It is well known that, in cancer, somatic mutations in *EGFR* lead to its constant activation with the result of an uncontrolled cell division [114]. On the opposite, germline *EGFR* missense variants, such as T790M, V834L and V843I, have been associated with rare cases of familial lung adenocarcinoma [115,116,117,118,119,120,121]. Additionally, a germline loss of function variant, affecting the extracellular domain of EGFR, has been described, but it was associated with a severe and lethal form of epithelial inflammation [122,123]. The c.3538_3541delGAAG variant in the *EGFR* gene, identified in one of our MBC patients, is located in the last exon, encoding the cytoplasmic domain with autophosphorylation function, but almost immediately after the last tyrosine (Y1173) subjected to phosphorylation [124]. The alteration determines the incorporation of 17 wrong amino acids, starting from position 1180, and a premature stop codon at position 1197. With the available information, it is not easy to predict the effect of this variant on the structure and function of the EGFR protein, but the variant has been already reported (rs781064539) [46] and is predicted to be deleterious according to the guidelines [39,48]. Of note, the variant carrier had a DCIS at 62 years old and also a non-Hodgkin lymphoma at 67 years old. The patient had no family history of cancer, so it was impossible to verify the segregation of the variant with the disease in his relatives. Consequently, further studies are needed to assess the real effect of the variant on the protein function and if it can have a role in cancer predisposition.

Finally, we did not identify any PV/LPV in 78.6% of the MBC patients subjected to the genetic test. This finding is compatible with the results obtained by other studies on MBC and, more in general, on genetic predisposition to cancer. We cannot exclude the presence of genetic alterations in regulatory regions or in other genes not analyzed in our panel. MBC, like other tumors, is a multifactorial disease and environmental/behavioral factors play a pivotal role in cancer development. Consequently, many of the MBC cases in which we did not find any genetic alteration could be the result of risk factors not linked to genetic predisposition.

## 5. Conclusions

Overall, our results confirmed the high risk of BC also for male carriers of *BRCA1/2* germline alterations, reinforced the emerging link between MBC and other genes involved in the predisposition to BC, and highlighted the association of novel genes with MBC.

## Figures and Tables

**Table 1:** List of the 94 cancer predisposition genes included in the Trusight Cancer panel.

Genes									
*AIP*	*ALK*	*APC*	*ATM*	*BAP1*	*BLM*	*BMPR1A*	*BRCA1*	*BRCA2*	*BRIP1*
*BUB1B*	*CDC73*	*CDH1*	*CDK4*	*CDKN1C*	*CDKN2A*	*CEBPA*	*CEP57*	*CHEK2*	*CYLD*
*DDB2*	*DICER1*	*DIS3L2*	*EGFR*	*EPCAM*	*ERCC2*	*ERCC3*	*ERCC4*	*ERCC5*	*EXT1*
*EXT2*	*EZH2*	*FANCA*	*FANCB*	*FANCC*	*FANCD2*	*FANCE*	*FANCF*	*FANCG*	*FANCI*
*FANCL*	*FANCM*	*FH*	*FLCN*	*GATA2*	*GPC3*	*HNF1A*	*HRAS*	*KIT*	*MAX*
*MEN1*	*MET*	*MLH1*	*MSH2*	*MSH6*	*MUTYH*	*NBN*	*NF1*	*NF2*	*NSD1*
*PALB2*	*PHOX2B*	*PMS1*	*PMS2*	*PRF1*	*PRKAR1A*	*PTCH1*	*PTEN*	*RAD51C*	*RAD51D*
*RB1*	*RECQL4*	*RET*	*RHBDF2*	*RUNX1*	*SBDS*	*SDHAF2*	*SDHB*	*SDHC*	*SDHD*
*SLX4*	*SMAD4*	*SMARCB1*	*STK11*	*SUFU*	*TMEM127*	*TP53*	*TSC1*	*TSC2*	*VHL*
*WRN*	*WT1*	*XPA*	*XPC*						

**Table 2:** Carriers of *BRCA1/2* pathogenic and likely-pathogenic variants.

Patient ID	Cancer	Age at Onset	Gene	Chr	cDNA (Transcript)	Protein	Variant Type	IARC Class [38]	dbSNP [46]	ClinVar [47]
A142	IDC	55y	*BRCA1*	17q21.31	c.-113-?_80+?del (NM_007294)	p.?	large deletion	5	–	–
A774	IDC	69y	*BRCA1*	17q21.31	c.4964_4982del (NM_007294)	p.Ser1655Tyrfs*16	frameshift deletion	5	rs80359876	pathogenic
TR140	IDC	57y	*BRCA1*	17q21.31	c.5266dupC (NM_007294)	p.Gln1756Profs*74	frameshift duplication	5	rs80357906	pathogenic
A379	IDC	58y	*BRCA2*	13q13.1	c.1238delT (NM_000059)	p.Leu413Hisfs*17	frameshift deletion	5	rs80359271	pathogenic
A581	IDC	77y	*BRCA2*	13q13.1	c.1813delA (NM_000059)	p.Ile605Tyrfs*9	frameshift deletion	5	rs80359306	pathogenic
T096	DCIS	68y	*BRCA2*	13q13.1	c.3195_3198delTAAT (NM_000059)	p.Asn1066Leufs*10	frameshift deletion	5	rs80359375	pathogenic
B156	IDC	64y	*BRCA2*	13q13.1	c.5073dupA (NM_000059)	p.Trp1692Metfs*3	frameshift duplication	5	rs80359479	pathogenic
A933	IDC	59y	*BRCA2*	13q13.1	c.6039delA (NM_000059)	p.Val2014Tyrfs*26	frameshift deletion	5	rs876660637	pathogenic
A98	IDC	56y	*BRCA2*	13q13.1	c.6039delA (NM_000059)	p.Val2014Tyrfs*26	frameshift deletion	5	rs876660637	pathogenic

IDC: infiltrating ductal carcinoma; DCIS: ductal carcinoma in situ; chr: chromosomal locus.

**Table 3:** Carriers of pathogenic and likely-pathogenic variants in genes other than *BRCA1/2*.

Patient ID	Cancer	Age at Onset	Gene	Chr	cDNA	Protein	Variant Type	IARC Class [38]	dbSNP [46]	ClinVar [47]
A841	IDC	38y	*ATM*	11q22.3	c.8319_8323dupTGTCC (NM_000051)	p.Pro2775Leufs*33	frameshift duplication	5	rs1555135596	pathogenic
A625	IDC	65y	*BAP1*	3p21.1	c.1110_1116delCATGCAG (NM_004656)	p.Met371Argfs*57	frameshift deletion	4	–	–
A512	IDC	36y	*CHEK2*	22q12.1	c.1100delC (NM_007194)	p.Thr367Metfs*15	frameshift deletion	5	rs555607708	pathogenic
A225	DCIS	62y	*EGFR*	7p11.2	c.3538_3541delGAAG (NM_005228)	p.Glu1180Profs*18	frameshift deletion	4	rs781064539	–
B887	IDC	75y	*PALB2*	16p12.2	c.73A>T (NM_024675)	p.Lys25*	nonsense variant	5	rs1248579792	pathogenic
A334	IDC	59y	*RAD51C*	17q22	c.181_182delCT (NM_058216)	p.Leu61Alafs*11	frameshift deletion	5	rs786203945	pathogenic

IDC: infiltrating ductal carcinoma; DCIS: ductal carcinoma in situ; chr: chromosomal locus.
